# Effects of Yu Linzhu on ovarian function and oocyte mitochondria in natural aging mice

**DOI:** 10.18632/aging.203626

**Published:** 2021-10-14

**Authors:** Zhen Yang, Mao-Lin Wei, Xiao-Ying Dong

**Affiliations:** 1 Department of Traditional Chinese Medicine, Institute of Traditional Chinese Medicine, Capital Medical University, Beijing, China

**Keywords:** Yu Linzhu, natural aging, ovarian function, oocytes, mitochondrial

## Abstract

Objective: To study the effect of Yu Linzhu on ovarian function and mitochondria in natural aging mice.

Methods: Female BALB/c mice were selected as normal group at 7–8 weeks and natural aging group at 9 months. The natural aging group was divided into Yu Linzhu intervention group and non-intervention group by intragastric administration once a day for 6 weeks. The morphology and blood flow of ovary were observed by ultrasound. Ovarian morphology and follicle were observed by HE staining. Hormone levels were analyzed by ELISA. Serum oxidative stress were detected by radioimmunoassay. The distribution of mitochondria in oocytes was observed by fluorescence staining. The ultrastructure of oocytes and the morphology of mitochondria were observed under electron microscope. The mitochondrial membrane potential was detected by JC-1.

Results: Two groups of aging mice had serious disturbance of estrus cycle. The ovarian area of the mice in the aging non-intervention group was smaller than that in the normal group, and the ovarian area of the mice in the aging intervention group recovered. The ovarian blood flow was weak or even disappear in the aging non-intervention group, and the blood flow in the intervention group was improved. The ovarian volume of mice in the non-intervention group was smaller than that in the normal group. Some ovarian tissues were adhered to the surrounding tissues. While in the intervention group, the ovarian volume increased, the degree of adhesion decreased, the infiltration of ovarian interstitial lymphocytes decreased, and the zona pellucida recovered. Granular cell arrangement returned neatly, egg cell shape recover regular and the number also increased. In the non-intervention group, E2 (Estrogen), AMH (Anti-Mullerian hormone) decreased (*P* = 0.0092 and *P* = 0.0334, respectively), FSH (Follicle stimulating hormone) increased (*P* < 0.0001). In the intervention group, FSH decreased (*P* = 0.0002), LH (luteinizing hormone) decreased and E2, AMH increased. In the non-intervention group, GSH-Px (Glutathione peroxidase) decreased (*P* = 0.0129), SOD (Superoxide dismutase) decreased, ROS (reactive oxidative species), MDA (Malondialdehyde) increased. In the aging intervention group, ROS, MDA decreased and GSH-Px increased. In the non-intervention group, mitochondrial expression was scattered at the concentrated distribution point, the length of mitochondria was mostly long and the average volume increased, the density decreased, the number decreased and some mitochondria fused, and lesions such as swelling, vacuolar degeneration and inclusion body formation, membrane potential decreased (*P* = 0.0002). In the aging intervention group, mitochondria were evenly distributed, the mitochondria were basically round, the distribution density was moderate, the inner ridge was clear, and the membrane potential of the aging intervention group increased.

Conclusion: Yu Linzhu can improve the ovarian function of natural aging mice by improving the mitochondrial function of oocytes.

## INTRODUCTION

Ovarian senescence is a biological process affected by many factors, gradually accumulating and continuously complicated, accompanied by the decrease of mitochondrial number, structural change and productivity of oocytes associated with the decrease of oxidative phosphorylation function. As the most important organelle in the cytoplasm, mitochondria helps oocyte maturation and embryonic development. Mitochondrial dysfunction and mitochondrial DNA mutation inhibit the normal development of oocytes. Mitochondria are the determinants of ovarian senescence [1, 2]. Changes in mitochondrial physiology and metabolism leading to excessive accumulation of reactive oxygen species are considered to be markers of aging [3]. Current studies have shown that age-related changes in mitochondria have a potential impact on oocyte quality in the study of age-related changes in the entire ovarian microenvironment [4].

Yu Linzhu is an ancient traditional Chinese medicine prescription that can treat infertility. It can warm spleen and kidney. In the formula, Four Gentlemen Decoction invigorate spleen and invigorate qi. Decoction of Four Ingredients nourish blood to fill essence. Semen Cuscute, Cortex Eucommiae and Cornu Cervi Dege Latinatum can warm and reinforce kidney yang. Pericardium Zanthoxyli can warm the body to promote follicle development and ovulation. The whole prescription promotes ovulation and ultimately improves ovarian function. Previous studies have found that Yu Linzhu can improve the serum hormone levels in POI patients [5]. Basic studies have further confirmed that it downregulates BAX/Bcl-2 inhibits the ovarian granulosa cell apoptosis [6]. Further studies have found that Yu Linzhu activates the mTOR pathway and promotes follicular development, which can improve ovarian dysfunction [7]. We speculate that it can improve ovarian senescence, so this experiment is to observe the effect of Yu Linzhu on ovarian function of natural aging mice, and to observe the effect of traditional Chinese medicine on ovarian function and oocyte mitochondria. Yu Linzhu can improve ovarian function by improving mitochondria.

## MATERIALS AND METHODS

### Animals

A total of 10 seven-to-eight-week-old SPF BABL/c female mice and 20 nine-month-old female mice were purchased from Beijing Vitonglida Experimental Animal Center (Animal Certificate No. SCXK2012-0001, Beijing, China). All mice were maintained at a constant temperature (20°C–22°C) and humidity (30%–70%) in a 12/12-hour light/dark cycle and had unrestricted access to water. All animal experiments were approved by the Ethics Committee of Animal Experiment, Capital Medical University, in accordance with the guidelines of the National Research for the Care and Use of laboratory animals.

### Main reagents

The following reagents were used: Serum follicle-stimulating hormone (FSH), anti-Muller tube hormone (AMH), luteinizing hormone (LH) and estradiol (E2) ELISA kits (Beijing Boorson Biotechnology Company). ROS, SOD, MDA, GSH-Px ELISA kits (Nanjing Jiancheng Biotechnology Company). 2 × HEPES buffer (Solarbio, H1080). 1 × PBS (Roby, RY-CC003a). Fetal bovine serum (Corning, 35-081-CV). Pregnant horse serum gonadotropin (PMSG, Solarbio, P9970). 10% chloral hydrate (Leagene, R00635-100 ml). Mito Traker Green FM (Invitrogen, M7514). 2.5% Gluta stationary liquid-electron microscope (Solarbio, P1126-100 ml). Swiss-Giemsa compound staining solution (Solarbio, G1020-100). Hoechst33342 staining solution (1 mg/ml) (Roby, RY-IF010a). Incomplete MEM (minimum essential medium, containing double antibodies, Kaiji Company, KGM41500-500). JC-1 (Biovision, 1130–5).

Traditional Chinese medicine preparation: Yu Linzhu ingredients include: angelica 12 g, ripe ground 12 g, dodder 12 g, ginseng 6 g, pepper 6 g, *Atractylodes macrocephala* 6 g, fried *Radix Paeoniae Alba* 6, *Eucommia ulmoides* 6 g, *Poria cocos* 6 g, antler cream 6 g, *Chuanxiong* 3 g, grilled licorice 3 g. Buy from Beijing Tongrentang Technology Co., Ltd. On the basis of the ‘human and animal body surface area equivalent dose scale’ decoction, it was determined that each mouse in the aging Chinese medicine group was given 0.468 g/ml, 0.3 ml/ Chinese medicine equivalent dose per day according to body weight, stored at 4°C refrigerators.

### Animal grouping and administration

10 female mice of adult reproductive age at 7–8 weeks were randomly selected as normal groups. Twenty aged female mice were randomly divided into 10 Yulinzhu intervention and 10 non-intervention groups. Animals in normal and non-interventional groups were given 0.3 ml saline solution once a day by intragastric administration once a day for 6 weeks. Yulinzhu intervention group was given 0.3 ml traditional Chinese medicine by intragastric administration once a day for 6 weeks.

### Vaginal smear

Small cotton swabs were soaked in saline, inserted into the vagina of mice approximately 5 mm, rotated clockwise 6 times, then rolled and smeared on a clean slide. The slide was naturally air-dried. The smears of vaginal secretions were collected for one week, stained with Swiss Giemsa staining solution for approximately 5 min, rinsed with water and observed under an ordinary optical microscope.

### High frequency small animal ultrasound system

The back fur of mice was removed with depilation ointment (Wei Ting), the mice were anesthetized by ultrasonic special table, and isoflurane (2.5%) was inhaled. A probe was placed 2–3 mm above the outer surface of the back of the mouse to see the appearance and disappearance of one side of the ovary as the moving area of the probe, and the other side of the ovary imaging was obtained by the same method. The color sampling frame was placed on the ovarian area corresponding to the back surface of the mouse to observe the arterial blood flow.

### Blood and tissue collection

The mice were anaesthetized by intraperitoneal injection of 4% chloral hydrate 0.1 ml/10 g. Take 10 ml 10% chloral hydrate plus 15 ml double-steamed water, mix evenly dilute to 4% chloral hydrate. After 5 minutes, the mice were anesthetized, then the blood was collected from heart. Then the animals were sacrificed by CO_2_ asphyxiation, the ovary and uterus were taken by routine aseptic method. One side of the ovary of three mice were fixed in precooled glutaraldehyde solution (2.5%), the other side of the ovary was fixed in formaldehyde. The formaldehyde-fixed ovarian tissue was dehydrated by gradient ethanol, paraffin-embedded, sliced, dewaxed, stained with conventional HE, and observed under a light microscope. The blood samples of the mice were allowed to stand and centrifuged, and ELISA were used to detect the contents of serum hormone and oxidative stress indexes in strict accordance with the kit instructions.

### Observation of oocyte mitochondria

#### Confocal microscopy

**Figure 1:**
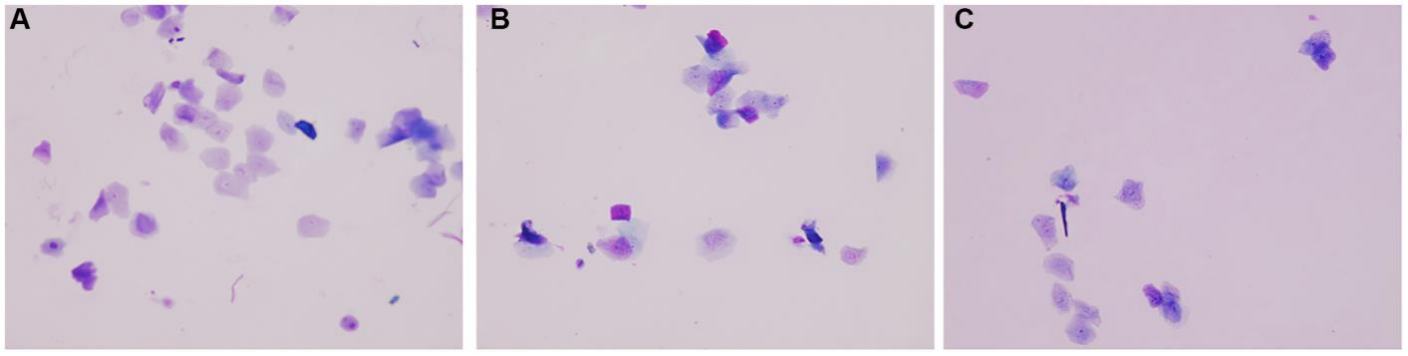
**Vaginal smear.** (**A**) Normal group; (**B**) Natural aging non-intervention group; (**C**) Natural aging intervention group (X20).

After 10/IU PMSG intraperitoneal injection of mice, they were killed by CO_2_ asphyxiation 48 hours later. Ovaries were cut off and placed in preheated HEPES, 5 for fluorescence and 5 for JC-1 (Figure 1). Under a microscope, the ovaries were released out of the ovaries with injection needle No. 5 and tweezers and punctured the follicles, and the oocytes were released from them. COC (Cumulus-Oocyte Complex) collected by oral straw was transferred to MEM medium containing 10% FBS and cultured in a 37°C gas incubator (5% CO_2_, 5% O_2_, 90% N_2_) for 17 hours to the MII stage. We did not choose HCG (human chorionic gonadotrophin) because it does not work, no follicles fall into the fallopian tube. To avoid mistakes, we chose to incubate directly. The oocytes that took off the granulosa cells were transferred to a 400 nmol/L Mito Tracker Green FM four-well dish preheated at 37°C and stained in a 37°C incubator for 30 minutes and washed with PBS 3 times for 5 minutes each time. After the above staining, the oocytes were added to 10 μg/ml Hoechst 33342, stained with nucleus for 10 minutes, and washed three times with PBS. The stained oocytes were transferred onto a slide for observation and analysis by laser confocal microscopy.

### Electron microscopy

The ovaries in Gluta fixation solution were cut into 1 × 1 × 1 mm^3^ sized tissue blocks, fixed in 4°C refrigerator for 4 hours, washed 3 times with 1XPB for 10 minutes each time, fixed with 1% osmotic acid for 1 h, washed 3 times for 10 minutes each time, and incubated with 2% U3HAC stain for 30 minutes. Then, the samples dehydrated in gradient alcohol (50%, 70%, 90%) for 10 minutes, 100% alcohol for 15 minutes, and 100% acetone for 15 minutes. Next, ultrathin slices with a thickness of 120 nm were prepared. The slices were stained with 4% UAC for 20 minutes and lead citrate for 5 minutes and observed with electron microscopy.

### Mitochondrial membrane potential detection

All MII stage oocytes were collected and transferred into 10 ug/ml JC-1 (Tetrachloro-tetraethyl benzimidazol carbocyanine iodide), stained for 25 min and washed 3 times with PBS. The stained oocytes were transferred onto slide and photographed under laser confocal. The fluorescence intensity of red and green light was determined by enzyme labeling instrument. The ratio of red to green fluorescence intensity represented the membrane potential. The red light excitation wavelength is 559 nm and emission wavelength 572 nm, the green light excitation wavelength is 488 nm and emission wavelength 520 nm.

### Statistical analysis

GraphPad Prism 8.0 software was used for analysis. The experimental data are represented by $\bar{X}\;\pm \text{ S}$, and the data were first tested for normal distribution. If the data were normally distributed, the differences between the two groups were compared by variance analysis, the variance was analyzed by the LSD method, and the variance was uneven by the Tamhane’s T2 method. If the data were not normally distributed, Kruskal-Wallis H test of multiple independent samples in nonparametric statistics was used. *P < 0.05* indicated that the difference was statistically significant.

## RESULTS

### Effect of Yu Linzhu on estrus cycle in mice

The observation of mouse vaginal smear showed that the estrus cycle of the two groups of aging mice was seriously disordered. By naked eye observation, it can be seen that the vaginal secretion of normal group mice is sticky and drawn, the vagina of both aging group mice is dry and astringent, there is no obvious vaginal secretion, and the mice of aging intervention group increase vaginal secretion. Under microscopic observation, it can be seen that the normal group mice were more homogeneous in the stage of estrus, and the keratinized epithelial cells were deciduous, and the keratinized epithelial cells in the aging non-intervention group were not uniform. The keratinized epithelium in the aging intervention group was better than that in the non-intervention group (Figure 1).

### Effect of Yu Linzhu on blood flow of mice

**Figure 2:**
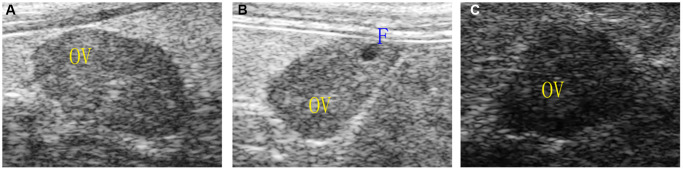
**Ovarian morphology under ultrasound.** (**A**) Normal group; (**B**) Natural aging non-intervention group; (**C**) Natural aging intervention group; OV, ovary; F, follicle.

**Figure 3:**
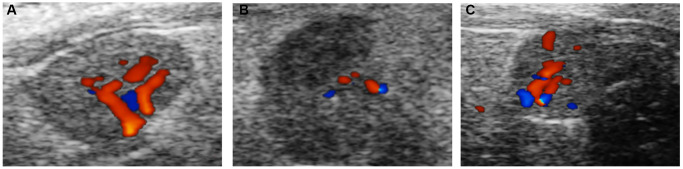
**Ovarian blood flow under ultrasound.** (**A**) Normal group; (**B**) Natural aging non-intervention group; (**C**) Natural aging intervention group.

The ovarian area of the mice in the aging non-intervention group was smaller than that in the normal group (*P = 0.0076*), and the ovarian area in the aging intervention group was larger than that in the non-intervention group (*P = 0.0210*) (Figure 2, Table 1). The ovarian blood flow in the normal group was rich, the mice in the aging non-intervention group were weak or even disappeared, and the blood flow in the intervention group was richer than that in the non-intervention group (Figure 3). Pulse pressure, pulse index and resistance index were higher in aging non-intervention group than in normal group. After drug administration intervention, pulse pressure and pulse index decreased (Table 2).

**Table 1:** Comparison of ovarian area in mice ($\bar{x}\;\pm \text{ s}$).

**Groups**	**Number**	**Normal group (mm^2^)**	**Natural aging non-intervention group (mm^2^)**	**Natural aging intervention group (mm^2^)**
area	5	5.41 ± 1.18	3.45 ± 0.36^★★^	5.11 ± 0.73^▲^

^★^*P* < 0.05 compared with normal group, ^★★^*P* < 0.01 compared with normal group, ^▲^*P* < 0.05 compared with non-intervention group, ^▲▲^*P* < 0.01 compared with non-intervention group.

**Table 2:** Comparison of blood flow in mice ($\bar{x}\;\pm \text{ s}$).

**Groups**	**Numbers**	**pulse pressure (=PS-LD) (mm/s)**	**PI**	**RI**
Normal group	5	16.03 ± 2.29	0.23 ± 0.04	0.20 ± 0.03
Natural aging non-intervention group	5	35.22 ± 21.43^★★^	0.55 ± 0.12	0.44 ± 0.08
Natural aging intervention group	5	28.50 ± 10.42^★^	0.43 ± 0.08	0.56 ± 0.13

^★^*P* < 0.05 compared with normal group, ^★★^*P* < 0.01 compared with normal group, ^▲^*P* < 0.05 compared with non-intervention group, ^▲▲^*P* < 0.01 compared with non-intervention group.

### Effect of Yu Linzhu on the morphology of mouse ovary

**Figure 4:**
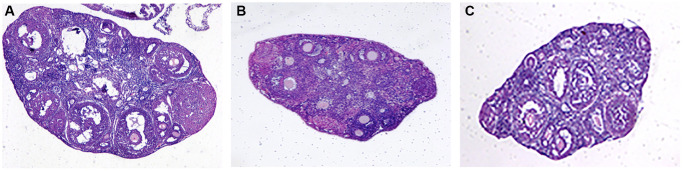
**Ovarian tissue sections.** (**A**) Normal group; (**B**) Natural aging non-intervention group; (**C**) Natural aging intervention group (X40).

Our naked eye observed that compared with the normal group, the ovarian volume of the mice in the aging non-intervention group was smaller than that in the normal group, and some ovarian tissues were adhered to the surrounding tissues, and the ovarian volume of the mice in the aging intervention group increased and the degree of adhesion was reduced. Under optical microscope, it was found that the ovarian interstitial lymphocytes infiltrated, some pellucida decreased, granulosa cells were arranged in disorder, oocytes morphology was deformed, narrowed or disappeared, and the infiltration of ovarian interstitial lymphocytes in mice in aging intervention group was alleviated. Particellucida decreased, granulosa cell arrangement recovered neatly, oocytes morphology recovered regularly, size recovered moderately, and number increased (Figure 4, Table 3).

**Table 3:** Comparison of follicle numbers in mice ($\bar{x}\;\pm \text{ s}$).

**Groups**	**Number**	**Normal group**	**Natural aging non-intervention group**	**Natural aging intervention group**
Follicle numbers	5	14.40 ± 2.79	5.80 ± 0.44^★★^	8.40 ± 1.140^★★^

^★^*P* < 0.05 compared with normal group, ^★★^*P* < 0.01 compared with normal group, ^▲^*P* < 0.05 compared with non-intervention group, ^▲▲^*P* < 0.01 compared with non-intervention group.

### Effect of Yu Linzhu on serum indicators

The E2, AMH decreased (*P = 0.0092 and P = 0.0334, respectively*) and the FSH increased (*P < 0.0001*) in the aging non-intervention group compared with the normal group. The FSH of aging intervention group decreased (*P = 0.0002*), the LH decreased and the E2, AMH increased compared with the aging non-intervention group (Table 4). The GSH-Px of aging non-intervention group decreased (*P = 0.0129*), SOD decreased and ROS, MDA increased compared with normal group. The aging intervention group ROS decreased and the GSH-Px increased compared with the aging non-intervention group (Table 5).

**Table 4:** Comparison of serum FSH, LH, E2, AMH expression levels in mice ($\bar{x}\;\pm \text{ s}$).

**Groups**	**Numbers**	**FSH (ng/mL)**	**LH (ng/mL)**	**E2 (pg/ml)**	**AMH (ng/mL)**
Normal group	5	2.82 ± 0.77	0.14 ± 0.07	8.32 ± 0.56	1.62 ± 0.81
Natural aging non-intervention group	5	5.31 ± 0.91^★★^	0.75 ± 0.21	7.13 ± 0.32^★★^	0.63 ± 0.30^★^
Natural aging intervention group	5	3.62 ± 1.24^▲▲^	0.35 ± 0.26	7.25 ± 0.11^★^	0.85 ± 0.39

^★^*P* < 0.05 compared with normal group, ^★★^*P* < 0.01 compared with normal group, ^▲^*P* < 0.05 compared with non-intervention group, ^▲▲^*P* < 0.01 compared with non-intervention group.

**Table 5:** Comparison of serum ROS, SOD, MDA, GSH-Px expression levels in mice ($\bar{x}\;\pm \text{ s}$).

**Groups**	**Numbers**	**MDA (nmol/ml)**	**ROS (U/ml)**	**SOD (U/ml)**	**GSH-Px (U/ml)**
Normal group	5	1.74 ± 0.42	20.64 ± 5.83	295.95 ± 24.20	1077.65 ± 260.45
Natural aging non-intervention group	5	2.85 ± 1.26	60.18 ± 19.70	229.94 ± 13.31	874.71 ± 234.36^★^
Natural aging intervention group	5	2.98 ± 0.52	45.57 ± 13.62	221.78 ± 35.90	940.59 ± 123.80

^★^*P* < 0.05 compared with normal group, ^★★^*P* < 0.01 compared with normal group, ^▲^*P* < 0.05 compared with non-intervention group, ^▲▲^*P* < 0.01 compared with non-intervention group.

### Effect of Yu Linzhu on mitochondria

**Figure 5:**
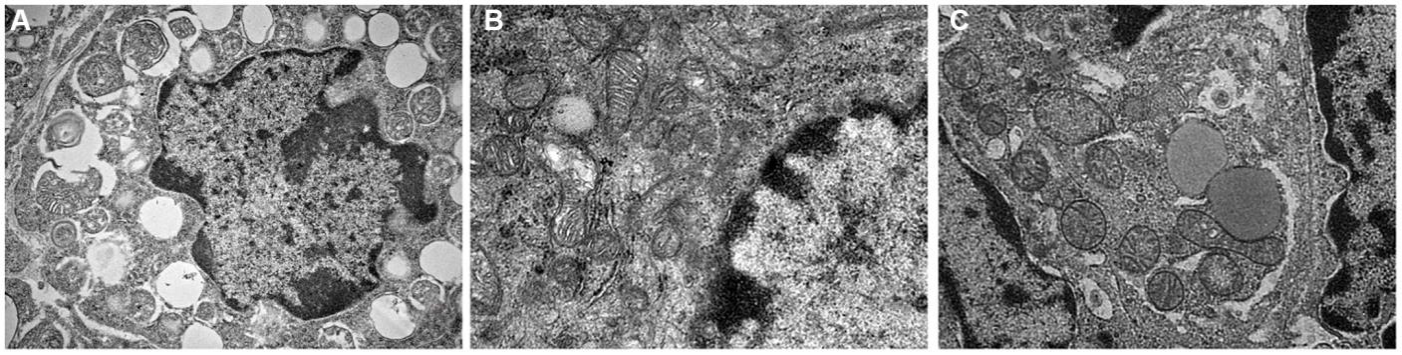
**Mitochondrial morphology under electron microscope.** (**A**) Normal group; (**B**) Natural aging non-intervention group; (**C**) Natural aging intervention group (X5000).

**Figure 6:**
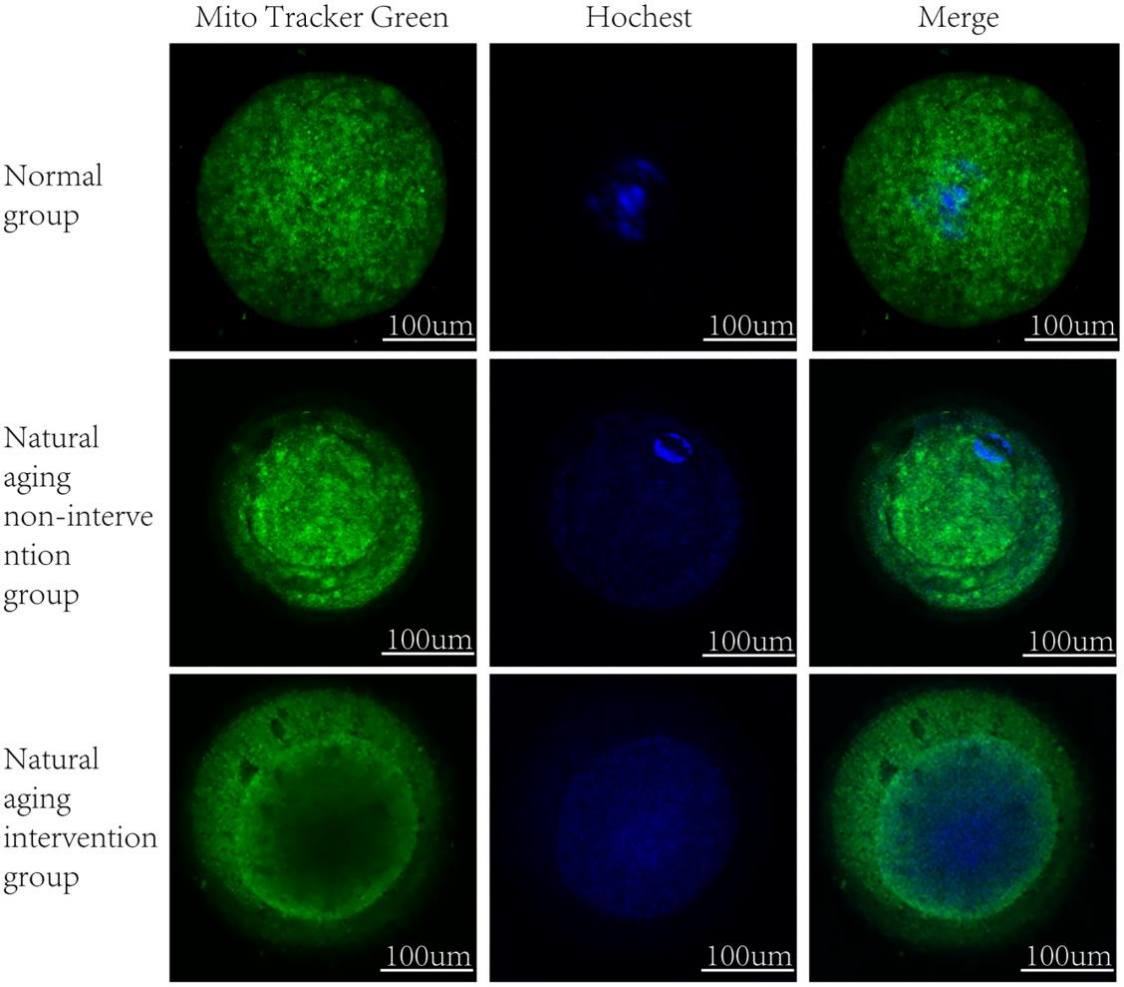
**Mitochondrial distribution.** MitoTracker Green staining mitochondria, Hoechst nuclear staining.

**Figure 7:**
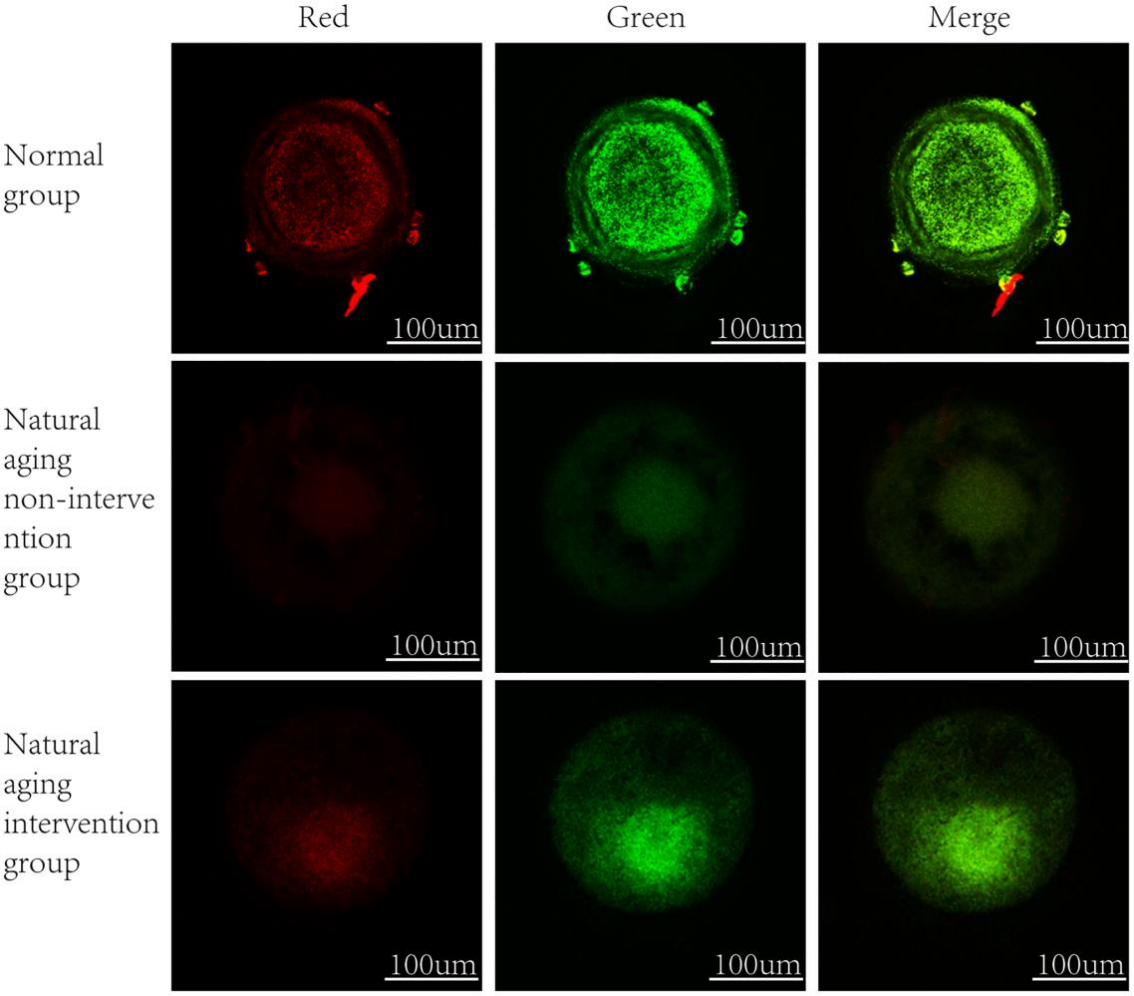
**Oocyte mitochondrial membrane potential.** In normal mitochondria, JC-1 gather in the mitochondrial matrix to form polymers, emit strong red fluorescence, unhealthy mitochondria due to the decline or loss of membrane potential, JC-1 in the form of monomers in the cytoplasm, produce green fluorescence.

It was observed by electron microscope that the mitochondria were mostly long and the average volume increased, the density decreased, the number decreased number of mitochondria was reduced, and the lesions such as swelling, vacuolar degeneration and inclusion body formation appeared. Compared with the non-intervention group, the mitochondria of the aging intervention group were basically round, the distribution density was moderate, and the inner ridge was clear (Figure 5). The expression of mitochondria in the aging group was scattered in the concentrated distribution point. Compared with the non-intervention group, the mitochondrial distribution in the aging intervention group was more uniform (Figure 6). Membrane potential decreased in aging group (*P = 0.0002*). Compared with the non-intervention group, the aging intervention group was higher (Figure 7, Table 6).

**Table 6:** Comparison of mitochondrial membrane potential levels in oocytes ($\bar{x}\;\pm \text{ s}$).

**Groups**	**Number**	**Normal group**	**Natural aging non-intervention group**	**Natural aging intervention group**
ΔΨm	5	1.05 ± 0.33	0.28 ± 0.02^★★^	0.40 ± 0.01^★★^

^★^*P* < 0.05 compared with normal group, ^★★^*P* < 0.01 compared with normal group, ^▲^*P* < 0.05 compared with non-intervention group, ^▲▲^*P* < 0.01 compared with non-intervention group.

## DISCUSSION

At present, the theory of oocyte aging mainly includes: aneuploidy theory, oxygen free radical-mitochondrial damage theory and so on. Mitochondria metabolize glucose into pyruvate and transport it to oocytes to generate ATP [8]. During the aging of higher organisms, the leakage of superoxide in respiratory chain and the damage cascade caused by reactive oxygen species, as well as mitochondrial metabolism during programmed cell death, have been the focus of research. A primordial follicle is the basic unit of germ cells. The follicle consists of oocytes and granulosa cells, including wall granulocytes and cumulus cells around oocytes (CCS). By secreting factors, oocytes upregulate the function of granulosa cells and improve the microenvironment of follicular and oocyte development ability [9]. Disordered follicles can lead to some reproductive problems, and increased ROS levels in oxidative stress can block the development of eggs in cystic follicles [10]. Ovarian senescence is mainly manifested in the decrease of oocyte quantity and quality, accompanied by the decrease of oocyte mitochondrial quantity, structural change and productivity. Mitochondria are the determinants of ovarian senescence. Oocyte quality is mainly changed by improving mitochondrial function and increasing the number of mitochondria [11]. Mitochondria are the main source of free radicals and ROS in cells. A large number of free radicals and ROS can lead to the further increase of ROS accumulation and mitochondrial DNA mutation, which leads to the exponential increase of mitochondrial mutation in malignant cycle. Then the synthesis of ATP decreased, resulting in cell cycle stagnation and even apoptosis. Mitochondria participates in the whole process of follicle development [12, 13]. De Bruin et al. found that mitochondrial fraction decreased and mitochondrial matrix density increased in the 38–45 age group [14]. ROS levels produced by mitochondrial respiratory function decreased with oocyte aging [15, 16].

In the theory of traditional Chinese Medicine, kidney dominates growth, development and reproduction. The Ming Dynasty Li Chan’s ‘Introduction to Medicine’ said: ‘People to middle age, kidney qi to deficiency.’ In the Ming Dynasty, Yu Tuan’s ‘Zhengzhuan of Medicine’ also said: ‘Abundant Kidney Qi brings longevity, kidney qi deficiency brings reduction in life-span.’ It can be seen that the aging of the human body begins with the aging of the kidney, and the life activities of the human body depend on the promotion of the kidney qi and essence. Once the kidney qi fails, the *five*-*Zang* also follow the failure. In the old age, the essence of the kidney is declining, which leads to the failure of other *Zang* functions, so it is necessary to take the method of tonifying the kidney and filling the essence and strengthening the life, so as to help generate qi and achieve the purpose of prolonging life and resisting aging.

In this study, the ovarian volume of aging mice was reduced and the blood flow decreased obviously by ultrasonic dynamic observation, which indicated that the decrease of blood flow was the characteristic of ovarian senescence. The ovarian blood flow recovered and improved ovarian function after Yulinzhu intervention in aging group. Morphological results also further confirmed that the infiltration of ovarian interstitial lymphocytes decreased after blood flow improvement, partial pellucida decreased, granulosa cell arrangement recovered neatly, egg cell morphology recovered regularly, size recovered moderately, and quantity increased. The further serum hormone level showed that E2, AMH increased and serum FSH, LH concentration decreased, indicating that hormone level improved. Also improved to delay ovarian aging, improve ovulation.

Based on this, we found that the fluorescence intensity of oocyte mitochondria in aging group was lower than that in blank group, and the mitochondrial distribution of Yulinzhu intervention group was more uniform. Morphologically, oocyte maturation process was accompanied by changes in mitochondrial distribution, and lack of mitochondrial redistribution was sign of insufficient cytoplasmic maturation [17]. The mitochondrial morphology was further observed by electron microscope. After intervention, the mitochondria in the aging group were basically restored to circle, swelling and vacuolar changes almost disappeared, and the inner ridge was clear, indicating that the recovery of mitochondrial structure was very important to slow down aging. Mitochondrial membrane potential is a sensitive indicator of mitochondrial damage, reflecting the functional metabolism of mitochondria. Finally, the mitochondrial membrane potential was detected and compared with the normal group, the membrane potential of the aging non-intervention group decreased. Compared with the non-intervention group, Yu Linzhu intervention group increased.

In conclusion, the decline of fertility in POI women is due to the decrease of follicle number and egg quality after the decline of ovarian function. Mitochondria such as quantity, distribution and membrane potential activate oocyte and embryo development. As the most important organelle of oxidation reaction, by improving ovarian microenvironment, enhancing ovarian blood flow and restoring ovarian function, mitochondrial vitality, follicle development and fertility will be improved. Further understanding the relationship between mitochondria and ovarian senescence and exploring the mechanism of traditional Chinese medicine to improve the quality of oocytes will help to provide a more optimized strategy for the prevention and treatment of female senescence and improve its pregnancy rate.
